# Photosensitivity and *CHD2* Variants

**DOI:** 10.15844/pedneurbriefs-29-9-1

**Published:** 2015-09

**Authors:** Rebecca García Sosa, Srishti Nangia

**Affiliations:** 1 Division of Neurology, Ann & Robert H. Lurie Children’s Hospital of Chicago, Chicago, IL; 2 Departments of Pediatrics and Neurology, Northwestern University Feinberg School of Medicine, Chicago, IL

**Keywords:** Eyelid Myoclonia with Absences, Photosensitive, Seizure

## Abstract

Investigators from multinational institutions hypothesized that disruption of *CHD2*, which encodes chromodomain helicase DNA-binding protein 2, would be associated with common forms of photosensitive epilepsy or photosensitivity manifesting as a photoparoxysmal response alone.

Investigators from multinational institutions hypothesized that disruption of *CHD2*, which encodes chromodomain helicase DNA-binding protein 2, would be associated with common forms of photosensitive epilepsy or photosensitivity manifesting as a photoparoxysmal response alone. They studied 580 patients with photosensitive epilepsy, defined as the presence of photoparoxysmal response with history of epilepsy or seizures reproducible by flickering lights. They also studied 55 patients with photoparoxysmal responses, but no seizures. *CHD2* sequencing was performed in this cohort and compared to a previously published cohort of 34,427 individuals, for which phenotypic data was not available. Unique *CHD2* variants were identified in 11 (11/1160 alleles; 0.95%) cases compared to 128 (128/68854 alleles; 0.19%) of controls, which suggests an over-representation in this population. Eyelid myoclonia with absences (EMA) had the highest frequency of unique variants (3/36 cases), more than expected for controls as a whole. Only one unique variant was identified in the group of photoparoxysmal responses without seizures. Investigators also studied functional consequences using *CHD2* loss in zebrafish. Morpholino injected larvae were thought to have more discharges and enhanced photoparoxysmal responses. Authors identify *CHD2* as a photosensitive epilepsy gene and an important contributor to both the absence seizures with eyelid myoclonia seizure type and eyelid myoclonia with absences epilepsy syndrome. [1]

COMMENTARY. Photosensitivity is an abnormal cortical response to flickering lights due to a genetically determined trait. When an electrographic correlate is identified it is known as a photoparoxysmal response. Photosensitive epilepsy, on the other hand is when the visual stimuli triggers seizures. [1] Photoparoxysmal responses have been described in certain epilepsy syndromes (Genetic Generalized Epilepsies, Eyelid Myoclonia with and without absence seizures, Dravet Syndrome, Myoclonic Atonic Epilepsy (MAE)), neurodegenerative diseases, and even normal individuals. [2] A more recent study attempted to assess the impact of *CHD2* mutations in a cohort of patients with MAE. [3] A *CHD2* mutation was found in 1/20 (5%) patients with Myoclonic Atonic Epilepsy. Photoparoxysmal responses and photoinduced seizures were described in this patient who was found to have a mutation not described in the cohort above. They concluded that although this gene might not be very significant in MAE, it may be responsible for generalized epilepsy with myoclonic-atonic and possibly atonic-myoclonic seizures, as well as intellectual disability and photosensitivity. As we continue to learn more about *CHD2* mutations, the different epilepsy phenotypes, and photoparoxsymal responses associated with this gene, we will learn more about the pathophysiology and perhaps be able to develop new target treatments for epilepsy.
